# Supplementary material for: Implication of thermal signaling in neuronal differentiation revealed by manipulation and measurement of intracellular temperature
Source: Nat Commun. 2024 May 9;15:3473. doi: 10.1038/s41467-024-47542-8 (PMC11082174; doi:10.1038/s41467-024-47542-8)
Supplement: Supplementary file 1 — Supplementary Information [file 41467_2024_47542_MOESM1_ESM.pdf]

Supplementary information for:

Implication of thermal signaling in neuronal differentiation revealed by manipulation and measurement of intracellular temperature

Shunsuke Chuma, Kazuyuki Kiyosue, Taishu Akiyama, Masaki Kinoshita, Yukiho Shimazaki, Seiichi Uchiyama, Shingo Sotoma, Kohki Okabe, Yoshie Harada

Supplementary Notes

Supplementary Note 1: Principles of the two types of intracellular fluorescent thermometers used in this study.
As shown in Figure 1d, FPTs, temperature-sensitive fluorescent polymers, undergo a structural change with increasing temperature, resulting in a change in fluorescence quantum yield and longer fluorescence lifetime due to the release of water molecules from inside the polymer. In this study, two kinds of FPTs with different temperature response ranges were used. Although a method for live-cell temperature imaging at 30 °C using FPTs has been established in previous studies[1,2], investigations of the cellular response to heating during neuronal differentiation should be performed at the physiological temperature of 37 °C, thus requiring the measurement of a higher temperature to determine heating intensity. Since conventional FPTs (hereafter referred to as FPT_{Low}) respond to temperatures of up to 40 °C, we newly synthesized FPT_{High} (Supplementary Figure 2), which responds to higher temperatures, using poly-*N*-isopropylacrylamide (NiPAM) as backbone. Both FPT_{Low} and FPT_{High} exhibited longer fluorescence lifetimes with increasing temperature (Figure 1f, Supplementary Figure 3). FNDs fluoresce due to the presence of a nitrogen–vacancy center (NVC) formed by nitrogen atoms and lattice defects (Figure 1e). Temperature-dependent electronic transitions based on the spin resonance of the NVC in FNDs can be measured by optically detected magnetic resonance (ODMR). As shown in Figure 1g and Supplementary Figure 4, the resonance frequency measured by ODMR decreases with increasing temperature.

Each of these thermometers has unique and outstanding performance in intracellular thermometry: FPTs are suitable to visualize intracellular temperature distribution because of their high intracellular dispersion and high sensitivity for temperature detection[3, 4] whereas FNDs are insensitive to the surrounding environment due to their rigid structure.

Supplementary Note 2: Influence of localized cytoplasmic heating on neurite outgrowth during NGF-mediated neuronal differentiation
In addition to nuclear heating, we examined the effects of temperature increase in the cytoplasm on neuronal differentiation. We heated the cytoplasm (ΔT = +3 °C) for 30 min and measured the length of neurites after 24 h of NGF treatment. No significant effect of cytoplasmic heating, measured in either condition (i) or (ii), on neurite

outgrowth was detected (Figure 3f, 3g, Supplementary Figure 5d, e). It should be noted that a moderate effect was observed when we compared neurite outgrowth after cytoplasmic heating in condition (ii) when only cells that showed protrusion elongation (i.e., excluding the 0-length data) were analyzed (Supplementary Figure 5f). These results suggested that the effects of cytoplasmic heating on neurite outgrowth are different from those of nuclear heating.

Supplementary Note 3: Intracellular temperature increase measured by PEI-FND during neuronal differentiation

PEI-FNDs (surface-modified FND with polyethyleneimine [PEI]) were incorporated into the cytoplasm of PC12 cells to measure the intracellular temperature using ODMR derived from electronic transitions based on the spin resonance of the NVC of the intracellular PEI-FND (Figure 5d). The resonance frequency of PEI-FNDs distributed inside the cell was significantly lower in differentiated cells compared with that in predifferentiated cells (Figure 5e) despite some variations due to the heterogeneity in particle size and NV density of individual FNDs[4]. This difference indicated an increase in intracellular temperature of approximately 0.6 °C. The difference in temperature increases measured by FPTs and FNDs may be due to the different intracellular distribution of these sensors as FPTs are distributed throughout the cytoplasm, whereas FND may be encapsulated in lipid bilayers in the cytoplasm[3]. Although the temperature dependence of the FND signal (i.e., magnetic resonance frequency) is small compared with the variation of the signal between particles, we used a chemical modification of the FND surface to control its localization (i.e., inside and outside the cell) to confirm that the detected signal derived from changes in intracellular temperature.

Supplementary Note 4: Suppression of the local intracellular temperature increase by large heat absorption of FPT

PolyNiPAM, the main backbone of FPT_{High}, exhibits large heat absorption capacity due to the phase transition that occurs above the lower critical solution temperature[5]. Taking advantage of this, we were able to suppress the local temperature increase by introducing a greater amount of FPT_{High} (1% w/v) into the cells (the intracellular concentration was estimated to be 0.04% w/v) than an amount (0.5% w/v) normally used for temperature measurement (Supplementary Figure 10). In the control experiment, a polyacrylamide (PAM) polymer[2] (control copolymer, CP: Supplementary Figure 11), which does not undergo phase transition in the temperature range investigated in this study (26 °C–43 °C), was injected.

Supplementary Figures

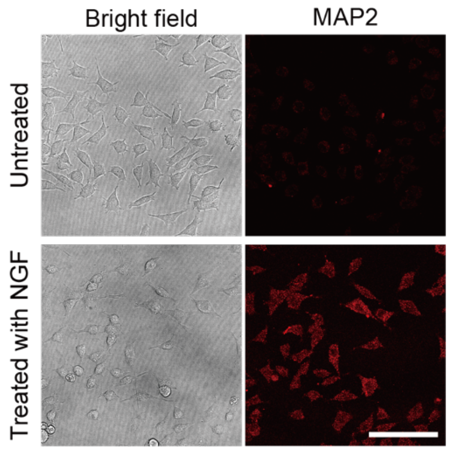

Supplementary Figure 1. Expression of MAP2 upon induction of neuronal differentiation by NGF. Bright field and immuno-fluorescence images of PC12 cells with or without 24 h of NGF treatment over two independent experiments. Scale bar represents 100 μm.

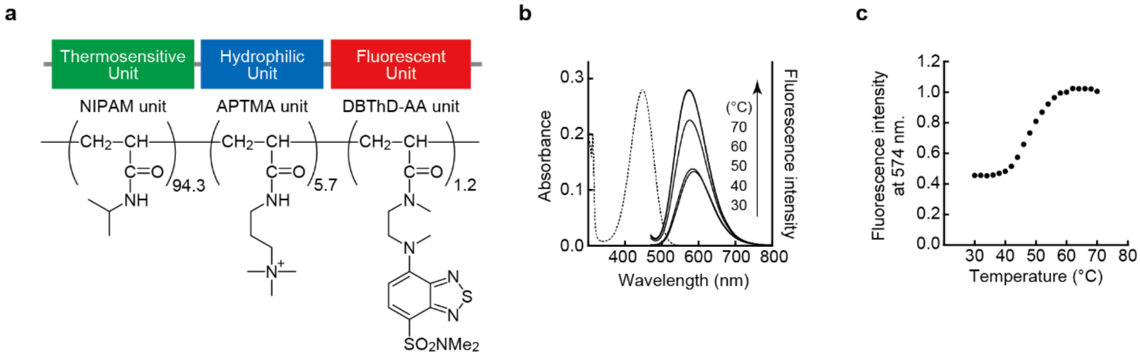

Supplementary Figure 2. Fluorescent polymeric thermometer with high-temperature phase transition (FPT_{High}). (**a**) Chemical structure. Thermosensitive unit: poly-*N*-isopropylacrylamide (NiPAM); hydrophilic unit: 3-(acrylamidopropyl) trimethylammonium (APTMA); fluorescent unit: (*N*-{2-[(7-*N*,*N*-dimethylaminosulfonyl)-2,1,3-benzoxathiadiazol-4-yl](methyl)amino}ethyl-*N*-methylacrylamide (DBThD-AA). Numbers indicate the proportion of each unit in the copolymer. (**b**) Absorption spectra in methanol (broken line, at 20 °C) and fluorescence spectra in a KCl solution (solid line). Fluorescence spectra were obtained with an excitation at 450 nm. (**c**) Fluorescence intensity response to the temperature variation in a KCl solution. Source data are provided as a Source Data file.

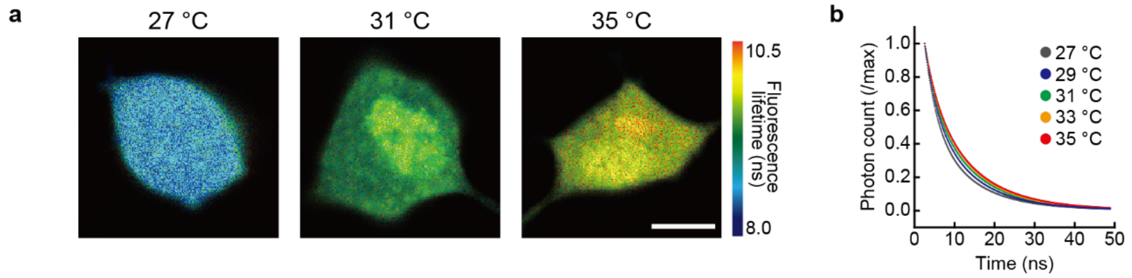

Supplementary Figure 3. Temperature-dependent fluorescence lifetime of FPT$_{\mathbf{Low}}$. (**a**) Fluorescence lifetime mappings in PC12 cells. Scale bar represents 10 µm. (**b**) Fluorescence decay curves in PC12 cells cultured at each temperature. Source data are provided as a Source Data file.

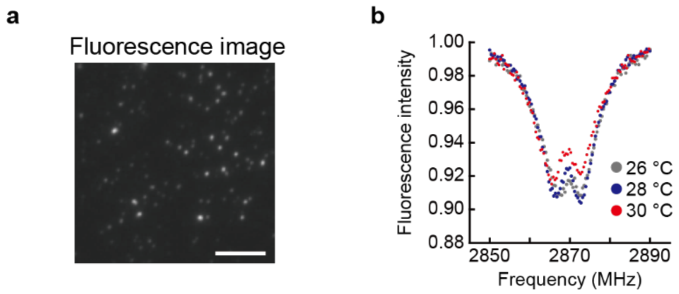

Supplementary Figure 4. Optically detected magnetic resonance (ODMR) spectrum of fluorescent nanodiamonds (FNDs). (**a**) Representative fluorescence image of polyethyleneimine (PEI)-FNDs on a coverslip. Scale bar represents 10 µm. (**b**) Representative ODMR spectra of FNDs at each temperature. Source data are provided as a Source Data file.

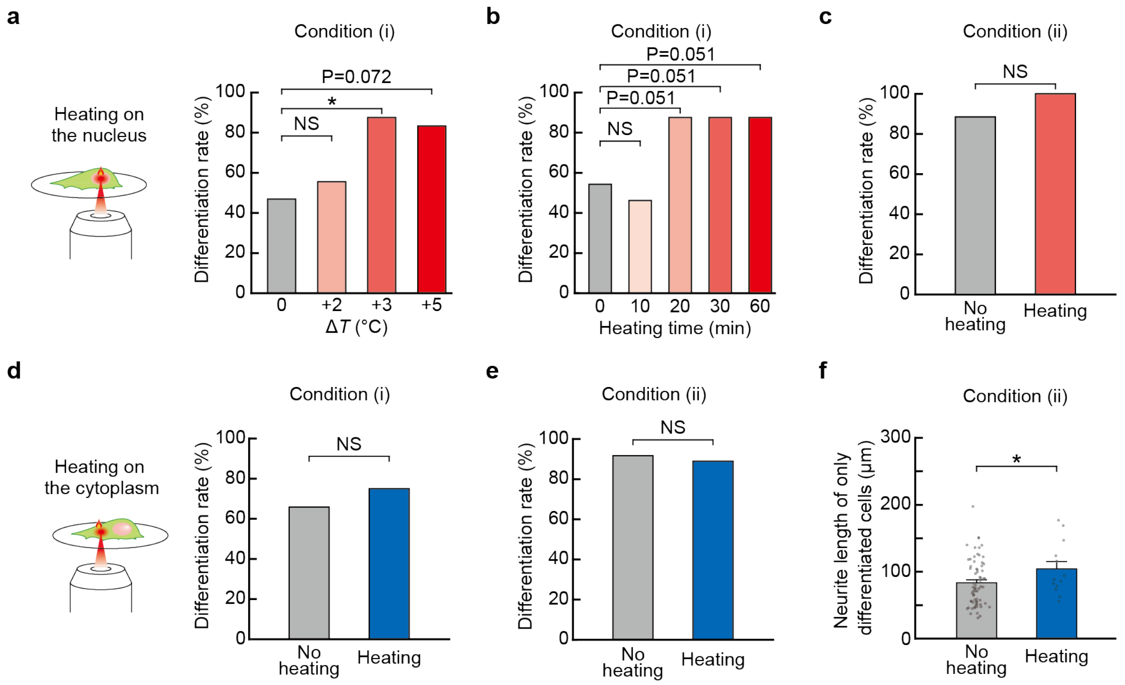

Supplementary Figure 5. Influence of intracellular local heating on neurite outgrowth rate during nerve growth factor (NGF)-induced neuronal differentiation of PC12 cells. (**a**) Influence of the intensity of IR laser heating (constant duration:1 h) on the differentiation rate after 24 h of NGF treatment. Data were acquired in condition (i) and are presented as means (n = 96 cells [no heating], 27 cells [ΔT = +2 °C], 8 cells [ΔT = +3 °C], and 6 cells [ΔT = +5 °C]). (**b**) Influence of the duration of IR laser heating (constant intensity: ΔT = +3 °C) on the differentiation rate after 24 h of NGF treatment in condition (i). Data are presented as means (n = 188 cells [no heating], 13 cells [heating time: 10 min], and 8 cells [heating time: 20, 30, and 60 min]). (**c**) Influence of nuclear heating (ΔT = +3 °C, heating time 30 min) on the differentiation rate after 24 h of NGF treatment in condition (ii). Data are presented as means (n = 27 cells [no heating] and 6 cells [heating]). (**d**) Influence of cytoplasmic heating (ΔT = +3 °C, heating time 30 min) on the differentiation rate after 24 h of NGF treatment in condition (i). Data are presented as means (n = 40 cells [no heating] and 11 cells [heating]). (**e**) Influence of cytoplasmic heating (ΔT = +3 °C, heating time 30 min) on the differentiation rate after 24 h of NGF treatment in condition (ii). Data are presented as means (n = 73 cells [no heating] and 15 cells [heating]). (**f**) Influence of cytoplasmic heating (ΔT = +3 °C, heating time 30 min) on the length of neurites after 24 h of NGF treatment in condition (ii). Data are presented as means ± standard errors of means (s.e.m.) (n = 67 cells [no heating] and 13 cells [heating]). Only data from cells with protrusion elongations in **Figure 3g** are plotted (the 0-length results were excluded). The number of independent experiments is the number of cells shown. $^{*}P < 0.05$, NS indicates not significant (one-sided binomial test in **a-e** and one-sided unpaired Student's t-test in **f**). Source data are provided as a Source Data file.

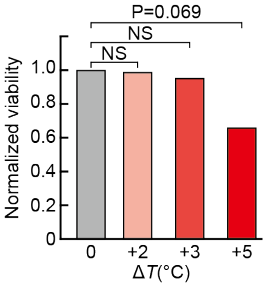

Supplementary Figure 6. Cell viability for the different temperature increases. The number of independent experiments is the number of cells shown (n = 137 [0 °C], 39 [+2 °C], 12 [+3 °C] and 13 [+5 °C] cells). NS indicates not significant (one-sided binomial test). Source data are provided as a Source Data file.

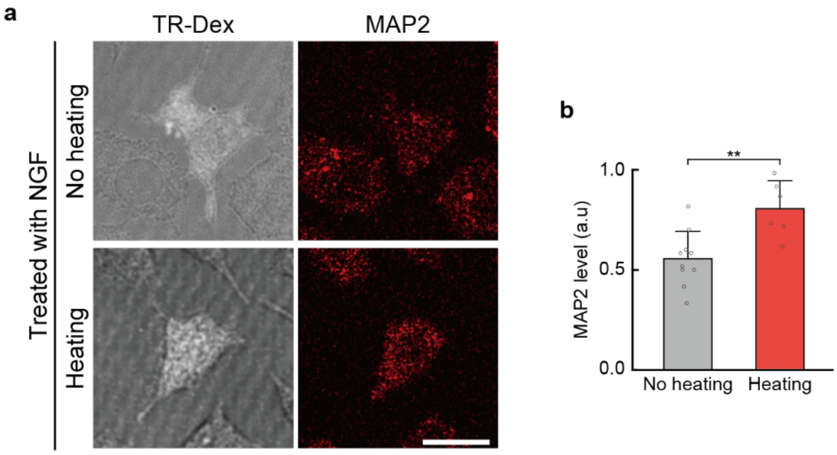

Supplementary Figure 7. Influence of nuclear heating on the expression of MAP2 during neuronal differentiation by NGF. (**a**) Bright field and immuno-fluorescence images of PC12 cells after 24 hours with and without heating immediately after NGF addition. Scale bar represents 25 μm. (**b**) Quantified MAP2 levels in cells with and without heating. Data are presented as means ± standard deviations (s.d.) (n = 10 [No heating] and 6 [Heating] cells). The number of independent experiments is the number of cells shown. **P < 0.01 (one-sided unpaired Student's t-test). Source data are provided as a Source Data file.

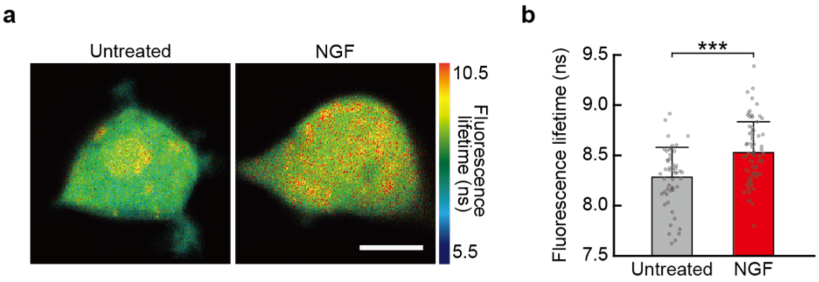

Supplementary Figure 8. Intracellular temperature measurement during neuronal differentiation using FPT$_{\mathbf{High}}$. (**a**) Intracellular temperature mapping before and after neuronal differentiation. Scale bar represents 10 µm. (**b**) Fluorescence lifetime in untreated cells or cells treated with NGF. Data are presented as means ±s.d. (n = 52 [untreated] and 72 [NGF] cells over two independent experiments). The temperature of the medium was maintained at 37 °C. $^{***}P < 0.001$ (one-sided unpaired Student's t-test). Source data are provided as a Source Data file.

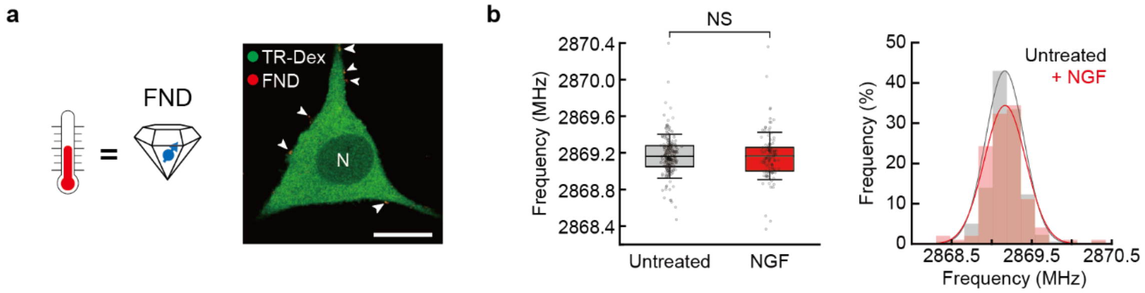

Supplementary Figure 9. Measuring the temperature of outer surface of cells during differentiation using unmodified FNDs. (**a**) Confocal fluorescence image of FNDs adhering to the cell membrane. Green and red are Texas Red-labeled dextran (TR-Dex, injected for visualizing cell morphology) and FNDs, respectively. White arrows indicate FNDs. Scale bar represents 10 µm. (**b**) Temperature measurement during neuronal differentiation using ODMR of FNDs. The scattered plot (left; data are presented as box and whiskers plots showing the median, mean [black dots], interquartile range, s.d.) and histogram (right) of resonance frequency of D (T) of FNDs in untreated (n = 203 particles in 13 cells over three independent experiments) and NGF-treated cells (n = 112 particles in 9 cells over five independent experiments). The temperature of the medium was maintained at 30 °C. NS indicates not significant (one-sided unpaired Student's t-test). Source data are provided as a Source Data file.

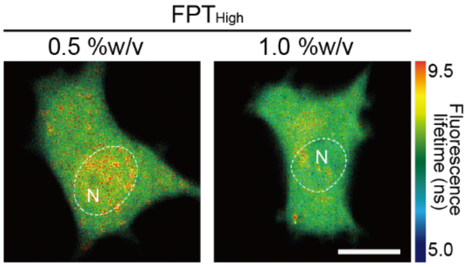

Supplementary Figure 10. Temperature mapping using normal (0.5% w/v) and high (1% w/v) concentrations of FPT_{High}. The temperature of the medium was maintained at 37 °C. Scale bar represents 10 µm.

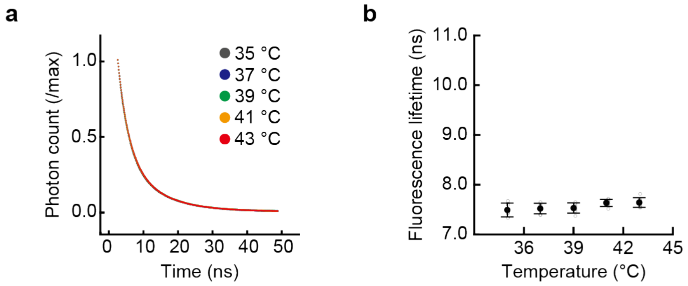

Supplementary Figure 11. Temperature-independent fluorescence lifetime of the control copolymer (CP) in PC12 cells. (**a**) Fluorescence decay curves of the control copolymer for each temperature. (**b**) Relationship between the fluorescence lifetime of the control copolymer and temperature. Data are presented as means ±s.d. (n = 6 [35, 39, 41 °C], 7 [37, 43 °C] cells over two independent experiments). Source data are provided as a Source Data file.

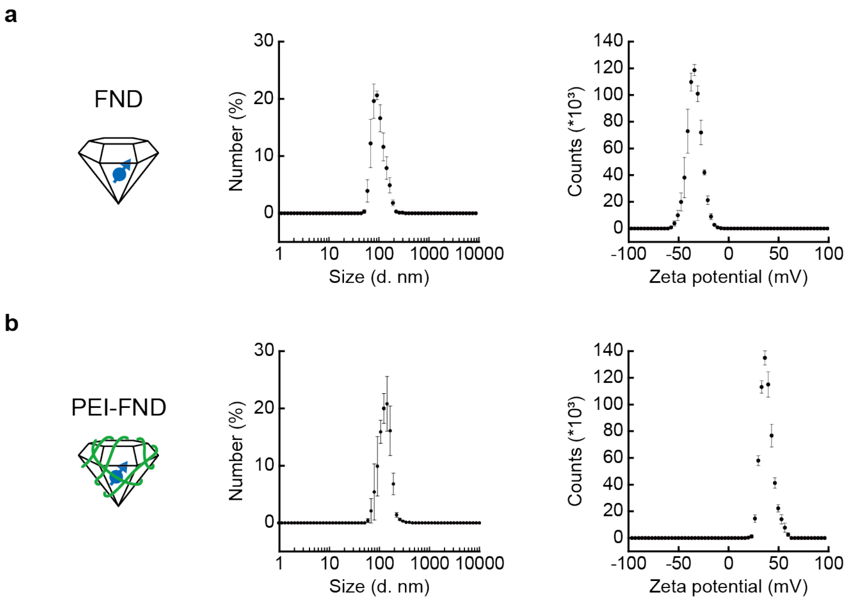

Supplementary Figure 12. Properties of unmodified and PEI-modified FNDs. (**a**) Histogram of diameter and zeta potential of unmodified FNDs. (**b**) Histogram of diameter and zeta potential of PEI-modified FND. (**a, b**) Data are presented as means ± s.e.m. (n = 3 independent experiments). Source data are provided as a Source Data file.

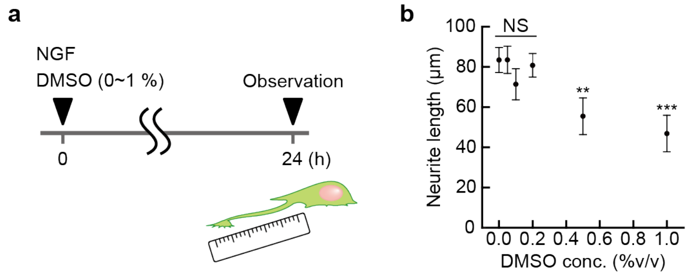

Supplementary Figure 13. Influence of dimethyl sulfoxide (DMSO) on neurite outgrowth in PC12 cells. (**a**) Experimental time flow. (**b**) Length of neurites after 24 h of NGF treatment as a function of DMSO concentration. Data are presented as means ± s.e.m. (n = 30 cells [0.05, 0.1, 0.2, 0.5, 1 %] and 31 cells [0 %] over two independent experiments). $^{**}P < 0.01$, $^{***}P < 0.001$ when compared with value obtained with 0% v/v DMSO. NS indicates not significant (one-sided unpaired Student's t-test). Source data are provided as a Source Data file.

Supplementary References

1. Okabe, K. *et al.* Intracellular temperature mapping with a fluorescent polymeric thermometer and fluorescence lifetime imaging microscopy. *Nat. Commun.* **3**, 705 (2012).
2. Hayashi, T., Fukuda, N., Uchiyama, S. & Inada, N. A cell-permeable fluorescent polymeric thermometer for intracellular temperature mapping in mammalian cell lines. *PLoS One* **10**, e0117677 (2015).
3. Sotoma, S. *et al.* In situ measurements of intracellular thermal conductivity using heater-thermometer hybrid diamond nanosensors. *Sci. Adv.* **7**, eabd7888 (2021).
4. Fujiwara, M. *et al.* Real-time nanodiamond thermometry probing in vivo thermogenic responses. *Sci. Adv.* **6**, eaba9636 (2020).
5. Ding, Y., Ye, X. & Zhang, G. Microcalorimetric investigation on aggregation and dissolution of poly(*N*-isopropylacrylamide) chains in water. *Macromolecules* **38**, 904–908 (2005).
