# Supplementary material for: Implication of thermal signaling in neuronal differentiation revealed by manipulation and measurement of intracellular temperature
Source: Nat Commun. 2024 May 9;15:3473. doi: 10.1038/s41467-024-47542-8 (PMC11082174; doi:10.1038/s41467-024-47542-8)
Supplement: Supplementary file 3 — Reporting Summary [file 41467_2024_47542_MOESM3_ESM.pdf]

nature portfolio

Corresponding author(s): Kohki Okabe
Last updated by author(s): March 26th 2024

Reporting Summary

Nature Portfolio wishes to improve the reproducibility of the work that we publish. This form provides structure for consistency and transparency in reporting. For further information on Nature Portfolio policies, see our Editorial Policies and the Editorial Policy Checklist.

Statistics

For all statistical analyses, confirm that the following items are present in the figure legend, table legend, main text, or Methods section.

n/a	Confirmed	
☐	☒	The exact sample size (*n*) for each experimental group/condition, given as a discrete number and unit of measurement
☐	☒	A statement on whether measurements were taken from distinct samples or whether the same sample was measured repeatedly
☐	☒	The statistical test(s) used AND whether they are one- or two-sided<br>*Only common tests should be described solely by name; describe more complex techniques in the Methods section.*
☒	☐	A description of all covariates tested
☒	☐	A description of any assumptions or corrections, such as tests of normality and adjustment for multiple comparisons
☐	☒	A full description of the statistical parameters including central tendency (e.g. means) or other basic estimates (e.g. regression coefficient) AND variation (e.g. standard deviation) or associated estimates of uncertainty (e.g. confidence intervals)
☐	☒	For null hypothesis testing, the test statistic (e.g. *F*, *t*, *r*) with confidence intervals, effect sizes, degrees of freedom and *P* value noted<br>*Give P values as exact values whenever suitable.*
☒	☐	For Bayesian analysis, information on the choice of priors and Markov chain Monte Carlo settings
☒	☐	For hierarchical and complex designs, identification of the appropriate level for tests and full reporting of outcomes
☒	☐	Estimates of effect sizes (e.g. Cohen's *d*, Pearson's *r*), indicating how they were calculated

Our web collection on statistics for biologists contains articles on many of the points above.

Software and code

Policy information about availability of computer code

Data collection	Confocal microscope images: Leica Application Suite LasX FLIM/FCS (version 3.5.6)<br>FLIM: Leica Application Suite LasX FLIM/FCS (version 3.5.6)<br>SymPhoTime 64 (Version 2.3)<br>ODMR: LabVIEW Development System (version 21.0)<br>Zetasizer Software (version 7.02)
Data analysis	Leica Application Suite X (Version 3.5.1)<br>SymPhoTime 64 (Version 2.3)<br>OriginPro 2022 (64-bit) SR1 9.9.0.225<br>Microsoft Excel 2021 (version 2304)

For manuscripts utilizing custom algorithms or software that are central to the research but not yet described in published literature, software must be made available to editors and reviewers. We strongly encourage code deposition in a community repository (e.g. GitHub). See the Nature Portfolio guidelines for submitting code & software for further information.

Data

Policy information about availability of data

All manuscripts must include a data availability statement. This statement should provide the following information, where applicable:

- Accession codes, unique identifiers, or web links for publicly available datasets
- A description of any restrictions on data availability
- For clinical datasets or third party data, please ensure that the statement adheres to our policy

All data that support the findings of this study are included in the paper. Microscopy data used for analyses are available from the corresponding author upon reasonable request. Requests will be fulfilled within 1 month. The data generated in this study are provided in the Source Data file. Source data are provided with this paper.

Research involving human participants, their data, or biological material

Policy information about studies with human participants or human data. See also policy information about sex, gender (identity/presentation), and sexual orientation and race, ethnicity and racism.

Reporting on sex and gender	N/A
Reporting on race, ethnicity, or other socially relevant groupings	N/A
Population characteristics	N/A
Recruitment	N/A
Ethics oversight	N/A

Note that full information on the approval of the study protocol must also be provided in the manuscript.

Field-specific reporting

Please select the one below that is the best fit for your research. If you are not sure, read the appropriate sections before making your selection.

☒ Life sciences ☐ Behavioural & social sciences ☐ Ecological, evolutionary & environmental sciences

For a reference copy of the document with all sections, see nature.com/documents/nr-reporting-summary-flat.pdf

Life sciences study design

All studies must disclose on these points even when the disclosure is negative.

Sample size	Sample sizes, provided in each figure legend, were based on previous reports (reference [34, 39] for intracellular temperature measurements and reference [42] for local intracellular manipulating).
Data exclusions	Cells that detached from the dish or displayed significant morphological alterations following microinjection, laser heating, or treatment with inhibitors were excluded from the analysis.
Replication	The experiments using cells were replicated to ensure reproducibility as follows; at least 2 independent experiments were performed, and key experiments were replicated more than three times independently.
Randomization	Randomization was not relevant because all cells were differently treated and analyzed in parallel to minimize experimental variation.
Blinding	All experiments were unblinded because these experiments are insusceptible to bias.

Reporting for specific materials, systems and methods

We require information from authors about some types of materials, experimental systems and methods used in many studies. Here, indicate whether each material, system or method listed is relevant to your study. If you are not sure if a list item applies to your research, read the appropriate section before selecting a response.

Materials & experimental systems

n/a	Involved in the study	
☐	☒	Antibodies
☐	☒	Eukaryotic cell lines
☒	☐	Palaeontology and archaeology
☐	☒	Animals and other organisms
☒	☐	Clinical data
☒	☐	Dual use research of concern
☒	☐	Plants

Methods

n/a	Involved in the study	
☒	☐	ChIP-seq
☒	☐	Flow cytometry
☒	☐	MRI-based neuroimaging

Antibodies

Antibodies used: We used a mouse monoclonal anti-MAP2 antibody (dilution 1:500) (M4403, Sigma-Aldrich) and a secondary anti-mouse IgG(H+L) antibody labeled with Alexa Fluor 647 (dilution 1:1000) (715-605-150, Jackson ImmunoResearch Laboratories) .

Validation: Anti-MAP2 antibody: the manufacturer's information confirms that this antibody is mouse IgG and has been shown to recognize MAP2 in rat, chicken, human, mouse, cow, and quail through WB analysis. Additionally, this antibody is suitable for staining formalin-fixed, paraffin-embedded sections.

Secondary antibody: the manufacturer's information confirms that this secondary antibody is donkey IgG and has been shown to specifically bind to mouse IgG(H+L) . The ELISA and/or solid-phase adsorption tests were conducted to ensure minimal cross-reaction with serum proteins from bovine, chicken, goat, guinea pig, Syrian hamster, horse, human, rabbit, and sheep.

Eukaryotic cell lines

Policy information about cell lines and Sex and Gender in Research

Cell line source(s): PC12 cells were acquired from RIKEN BioResource Center.

Authentication: PC12 cell line was not authenticated.

Mycoplasma contamination: We checked that the cell line was tested negative for mycoplasma by MycoStrip (rep-mys-10, InvivoGen).

Commonly misidentified lines (See ICLAC register): No commonly misidentified line was used.

Animals and other research organisms

Policy information about studies involving animals; ARRIVE guidelines recommended for reporting animal research, and Sex and Gender in Research

Laboratory animals: Mouse, C57BL/6J, wild type, embryonic 14–15-day

Wild animals: No wild animal was used in this study.

Reporting on sex: Both sexes pups were used without distinction.

Field-collected samples: No field collected samples were used in this study.

Ethics oversight: Preparation of primary mouse cortical neurons was performed in accordance with the animal health care guideline of Japan and approved by the ethics committee of AIST (permission No. 2023-0008).

Note that full information on the approval of the study protocol must also be provided in the manuscript.
